# Mechanical Behavior Analysis of Polypropylene-Based Composites and a Photopolymer Resin via Tensile and Scratch Testing

**DOI:** 10.3390/polym17162180

**Published:** 2025-08-09

**Authors:** Sergiu Gabriel Pal, Viorel Goanta, Ciprian Ionut Moraras, Vlad Carlescu

**Affiliations:** Mechanical Engineering, Mechatronics and Robotics Department, Mechanical Engineering Faculty, Gheorghe Asachi Technical University of Iasi, 700050 Iasi, Romania; sergiu-gabriel.pal@student.tuiasi.ro (S.G.P.); ciprian-ionut.moraras@academic.tuiasi.ro (C.I.M.); vlad.carlescu@academic.tuiasi.ro (V.C.)

**Keywords:** plastic properties, mechanical behavior, scratch test, tensile test, photopolymer resin, polypropylene-based composites

## Abstract

This study investigates the mechanical behavior of various plastic materials through tensile and scratch testing. Three polypropylene-based composites—PP-GB30GF10, PP-TD40, and PP-GF20—were subjected to uniaxial tensile tests in accordance with standard protocols to assess their strength, stiffness, and elongation characteristics. The results highlight notable differences in the tensile performance depending on the type and percentage of reinforcing fillers, such as glass fibers and talc. In parallel, the scratch resistance was evaluated for specimens produced via stereolithography (SLA) using Formlabs Black V4 resin, a common photopolymer used in prototyping applications. The scratch test aimed to characterize the surface durability under localized mechanical stress. The findings contribute to a better understanding of the mechanical performance of these materials and their potential applications in fields requiring both structural integrity and surface resilience, such as automotive components and functional prototyping.

## 1. Introduction

The selection of suitable polymeric materials for engineering applications often requires a comprehensive understanding of their mechanical behaviors under various types of loading and environmental conditions. Thermoplastics, particularly polypropylene (PP)-based composites, are widely used in industries such as automotive, consumer goods, and packaging due to their favorable balance of mechanical properties, low density, ease of processing, and cost-effectiveness. To further enhance their performance, PP is commonly reinforced with fillers, such as glass fibers or talc, which significantly influence properties like stiffness, strength, and impact resistance.

Evaluating the mechanical behavior of such materials typically involves tensile testing, which provides critical information regarding their elastic modulus, tensile strength, and elongation at break. In this context, materials such as PP-GB30GF10 (30% glass beads and 10% glass fiber), PP-TD40 (40% talc-filled), and PP-GF20 (20% glass fiber) offer diverse mechanical responses depending on the filler type and concentration.

Simultaneously, with the increasing use of additive manufacturing for rapid prototyping and small-scale production, understanding the surface durability of photopolymer resins has become equally important. Resins, such as Formlabs Black V4, used in stereolithography (SLA) 3D printing are commonly applied in applications where fine surface detail and moderate mechanical performance are required. However, their scratch resistance—a key indicator of surface wear and aesthetic longevity—remains a critical factor for functional applications.

This study aims to assess and compare the mechanical performance of several polypropylene-based composites through tensile testing, as well as to evaluate the scratch resistance of a 3D-printed photopolymer resin specimen. The findings are intended to inform material selection for applications requiring both mechanical robustness and surface durability.

Polypropylene (PP) continues to be one of the most widely used thermoplastics in the automotive, packaging, and electronics industries due to its favorable balance of mechanical properties, cost-efficiency, chemical resistance, and ease of processing [1,2]. However, its relatively low scratch resistance and moderate stiffness, particularly in applications requiring mechanical durability and surface integrity, have driven the extensive use of reinforcements and fillers—most notably glass fibers (GFs), talc, and other mineral or hybrid additives [3,4,5].

The enhancement of PP’s mechanical properties through the incorporation of fillers, such as glass fiber (GF), talc, and calcium carbonate (CaCO_3_), has been the subject of broad scientific and industrial interest [4,6,7,8]. These fillers significantly alter the mechanical response under tensile loading and scratching, not only by increasing stiffness and strength but also by influencing failure mechanisms and surface wear resistance [9,10]. Among these, short glass fibers (SGFs) are particularly effective at enhancing tensile properties, while talc contributes more significantly to scratch and mar resistance due to its platy morphology [1,3,9]. Furthermore, the orientation of fibers or platelets in the PP matrix—especially in injection-molded parts—affects the anisotropic nature of both strength and scratch resistance, emphasizing the need for directionally resolved testing [2,11,12].

In the current study, we investigate three representative polypropylene-based composite formulations: PP-GB30GF10, PP-TD40, and PP-GF20, which incorporate glass beads, talc, and varying contents of glass fiber, respectively. These materials reflect a cross-section of commercially relevant PP composites designed for automotive and structural applications, with each possessing distinct mechanical profiles due to differences in the filler type, morphology, and concentration. For example, PP-GB30GF10 benefits from combined reinforcement from spherical and fibrous inclusions, while PP-TD40 is optimized for high rigidity via talc loading. PP-GF20 serves as a mid-range formulation emphasizing balanced strength and toughness [4,6,13].

The tensile properties of PP composites are strongly dependent on filler type and fiber alignment. As shown by Chiou and Lin [7], short glass fibers improve tensile strength and modulus, especially when aligned along the loading direction. Talc-filled systems, while enhancing stiffness and scratch resistance, typically result in reduced ductility and impact performance due to stress concentrations at the matrix–filler interface [3,14,15]. Therefore, our investigation includes tensile testing across samples with different fiber/filler orientations, aiming to elucidate the anisotropic mechanical response inherent in these composites.

Scratch resistance is another crucial criterion, especially for visible or high-contact components in consumer goods and automotive interiors. The scratch behavior of PP has been widely characterized under both constant and progressive load conditions [1,4]. Fillers influence the scratch resistance through a combination of hardness modification, surface crystallinity, and crack deflection mechanisms [10,15,16]. For instance, Chu et al. [9,10] and Xiang et al. [1,16] observed that mineral fillers, such as talc, improve the resistance to scratch initiation and propagation by increasing the composite hardness and altering plastic deformation zones beneath the surface. The use of additives like PDMS has also been shown to reduce friction and improve mar resistance [17,18], though such modifications were not the focus of the present study.

To capture surface-level damage characteristics, we also include scratch testing of a photopolymer resin, specifically a black V4 resin used in the 3D printing of electronic housings. Photopolymer resins are increasingly employed in additive manufacturing due to their precision and surface quality but often suffer from brittle behavior and poor scratch resistance [5,12]. Although not directly comparable with semi-crystalline PP composites, the resin serves as a baseline for evaluating performance differences in functional components fabricated via 3D printing. Work by Cuesta et al. [5] and Fidan et al. [12] has underscored the mechanical limitations of photopolymers in scratch and fracture testing, particularly under multidirectional loading or indentation stresses.

Given the rising integration of hybrid fillers and the pursuit of mechanical and aesthetic performance in polymer systems, numerous studies have investigated the synergistic effects of dual- or triple-reinforcement systems [4,6,8,19]. For example, Soy et al. [4] demonstrated that combinations of GF, talc, and CaCO_3_ in recycled PP not only enhanced the stiffness but also improved the wear and scratch resistance. The hybridization of reinforcements not only broadens the property envelope but also enables tuning of specific attributes such as the fracture toughness, surface integrity, and thermal stability [20,21,22].

Study [23] investigated polypropylene copolymer composites reinforced with calcium silicate, focusing on their mechanical strength and scratch resistance. They report substantial improvements in hardness, stiffness, and scratch scores due to the platy calcium silicate particles. Under scratch testing, these composites exhibited smaller and shallower scrapes compared with unfilled PP, showing the filler’s effectiveness in distributing stress and hindering crack propagation under progressive loading.

Deniz V. [24] studied polystyrene nanocomposites with various nano-fillers—spherical (SiO_2_, Al_2_O_3_), tubular (halloysite), platy (mica), and fibrous (multi-walled carbon nanotubes, CNTs). They showed that rigid nanofillers can increase the elastic modulus by up to 80% (especially carbon nanotubes), and the strength at break by ≈20% for carbon black additions. Notably, nonlinear elastic properties (e.g., third-order moduli) are more sensitive to filler presence than linear ones, offering insights into composite toughness and deformation mechanisms.

However, despite the extensive literature, gaps remain in the direct comparison of these composites under aligned mechanical and tribological protocols, particularly with fiber orientation control. Additionally, the effect of printing technology and resin formulation on scratch resistance in additive manufacturing components remains underexplored. Our study bridges these areas by conducting tensile testing across directional specimens and performing standardized scratch tests on molded PP composites and printed photopolymer resin components.

The novelty of this work lies in the dual characterization of both mechanical strength and scratch resistance across conventionally reinforced PP composites and a 3D-printed photopolymer, evaluated under realistic loading and use conditions. By correlating the filler content, orientation, and morphology with performance outcomes, the findings aim to assist in material selection and design optimization for applications where mechanical durability and surface aesthetics are both essential.

This paper is organized as follows: Section 2 describes the materials and methodologies employed, including sample preparation, tensile testing conditions, and scratch protocols. Section 3 presents the experimental results and discusses key observations on the impact of the filler orientation, resin performance, and scratch behavior. Section 4 concludes with final remarks and design implications.

Polypropylene (PP) is widely used in engineering applications due to its favorable mechanical properties, chemical resistance, and cost-effectiveness. Reinforcement with fillers, such as talc or glass fibers, is a common method for enhancing specific mechanical behaviors. Talc fillers tend to improve stiffness, while glass fibers contribute to improved tensile and flexural strength due to their high aspect ratio and load-bearing efficiency (1–3).

Meanwhile, photopolymer resins used in SLA 3D printing offer precision and geometric freedom but exhibit different mechanical behavior due to layer-wise deposition and curing. One limitation of SLA parts is anisotropy, particularly due to weak inter-layer bonding, which can affect the tensile and scratch resistances (4–6).

This study aims to evaluate and compare the mechanical behavior of three PP composites—PP-GB30GF10, PP-TD40, and PP-GF20—and a commercial SLA photopolymer (Black V4 resin). The hypotheses guiding this work are as follows:

**H1:**  *PP-GF20 will exhibit the highest tensile strength due to the directional reinforcement of glass fibers*.

**H2:**  *PP-TD40 will demonstrate a higher stiffness but lower ultimate strength due to talc’s non-fibrous morphology*.

**H3:**  *For the SLA resin, samples aligned with the print layers (horizontal) will perform better at scratch resistance due to stronger in-plane cohesion*.

By correlating the mechanical performance with material formulation and sample orientation, this study contributes practical data for design decisions involving molded and 3D-printed components.

## 2. Materials and Methods

**Figure 1:**
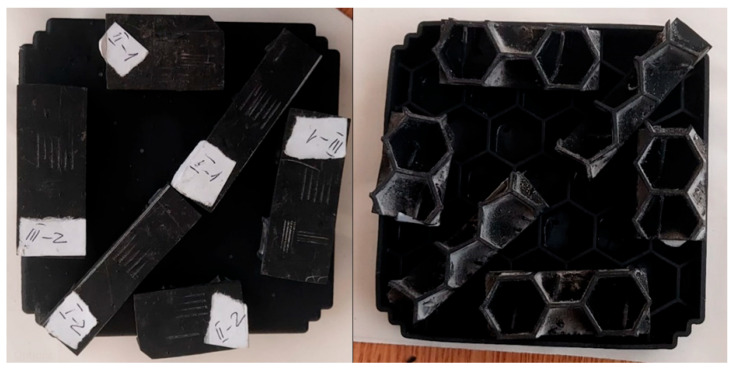
Test probes for scratch tests extracted from reference housing.

To study the resin Black V4, we performed a test with samples extracted from a 3D-printed housing. The scope for this test is to study the layer orientation to identify the best resistance for this part; this is why we cut one housing and extracted test samples for the scratch test that have different layer orientations. The SLA-printed housing offered the best support for our scratch test equipment due to the honeycomb reinforcement ribs; in Figure 1, the test probes that we extracted from it are visible.

The test probes were labeled with the following characters: I_1, I_2, II_1, II_2, III_1, and III_2, resulting in a total of 6 test samples. Depending on the layer orientation, we can observe 3 different types of test probes: I—diagonal layer orientation, II—transversal layer orientation, and III—perpendicular layer orientation.

The scratch resistance testing was performed using a Tribometer UMT-2 (CETR, now Bruker) sourced from Campbell, California, USA. A blade pointer (90° tip, 0.4 mm radius) applied a progressive load (1–20 N) across a 50 mm track. The critical load (Lc) at which visible surface damage or delamination occurred was recorded for each orientation. This equipment serves to measure mechanical and tribological properties of different metals, plastic parts, ceramic parts, and different alloys with different thicknesses, but also oils, lubes, and other types of grease.

**Figure 2:**
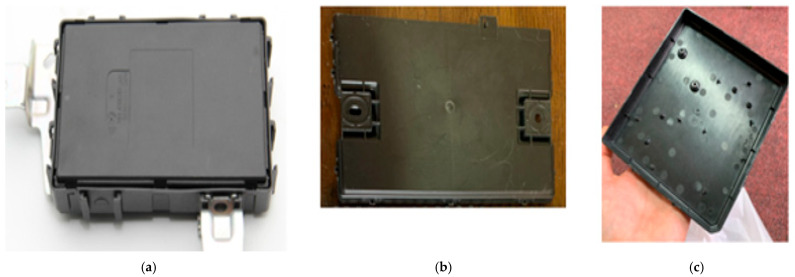
Plastic housings made from polypropylene with different fillers. (**a**) Housing 1; (**b**) Housing 2; (**c**) Housing 3.

For the tensile test, we had three different plastic housings with similar exterior dimensions as the base, with similar overall material thicknesses and one single injection mold point placed in the middle of the housing. Below are the materials for each housing:

1. Housing 1 (Figure 2a)—material PP-GB30GF10 (polypropylene, 30% glass beads + 10% glass fibers) with traction resistance of 44.1 MPa;
2. Housing 2 (Figure 2b)—material PP-TD40 (polypropylene, 40% talc-filled) with traction resistance of 32 MPa;
3. Housing 3 (Figure 2c)—material PP-GF20 (polypropylene, 20% glass-fiber-reinforced) with traction resistance of 80 MPa.

**Figure 3:**
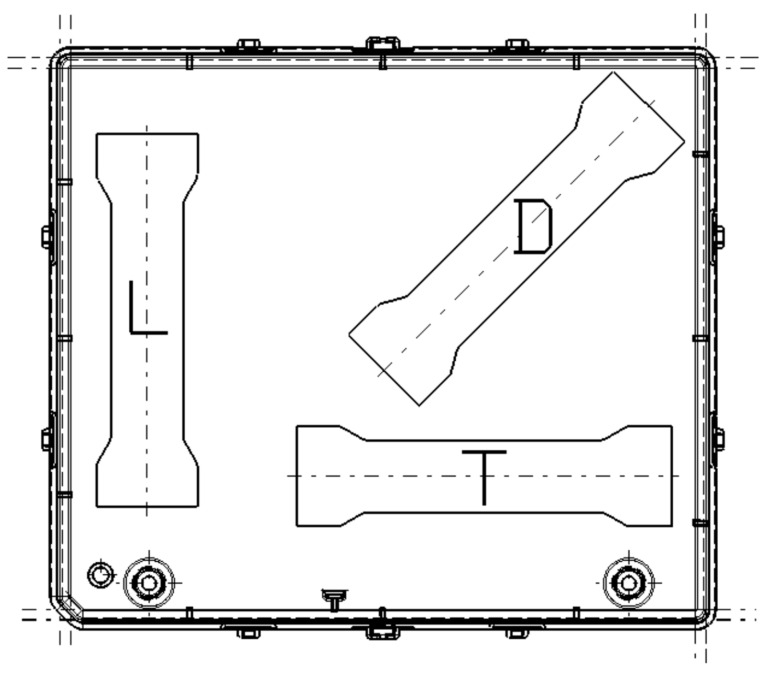
Example of the distribution for test probes on a housing.

In these tests, we wanted to cut three test probes from each housing (similar to the situation in Figure 3 with longitudinal, transversal, and diagonal components by considering the length of the housing and arranging around the injection point) from the flat area of each housing. The purpose of this test was to study the effect of the distribution of additives (fibers and minerals) in the plastic material. Given the radial spread of the fibers in relation to the injection point, the three test probes registered different values for the tensile test.

To perform the tensile test, a testing machine INSTRON 8801 from Illinois Tool Works (ITW) sourced from Darmstadt (Germany) was used, along with a strain indicator and recorder model VISAHY P3 from Vishay Precision Group (VPG) sourced from Heilbronn (Germany), and each test probe had a precision sensor type WA-06-030WT-120, also from VPG, attached to it. The test regime was established by increasing the deformation rate to a value of 0.5 mm/min. The results obtained from the test are displayed on the screen, extracted in the format delivered by the testing machine software WaveMatrix version 1.9, and then processed in Excel in the form of graphs.

Bidirectional electro-strain gauge rosettes of the type WA-06-030WT-120 were mounted with attached cables, and each brand had an electrical resistance of 120 Ω. To apply these transducers, the surface was wiped with a cotton cloth soaked in ethyl alcohol. The rosettes were glued using the adhesive Z-70 (cyanoacrylate) from HBM, with its polymerization being accelerated with an M-Bond 200 Catalyst C from Vishay.

In Table 1 is a summary table of all samples used in tensile and scratch tests. Goal for this table is to clarify the meaning of letters from Figure 3 and more aspects related to orientation of the cut samples for scratch tests.

## 3. Results

### 3.1. Scratch Test

The test probes were fixed on the linear table of the tribometer using metal supports fixed with screws and the scratch test was conducted using a pointer made of tungsten carbide with a 0.4 mm radius at the tip. The pointer was fixed on an elastic adaptor, which allowed movements in the vertical direction, making it possible to adapt the load force measured by the sensor up to 20 N. Six samples were prepared from SLA-printed housings in Formlabs Black V4 resin. Two samples were cut per orientation (H, V, D), corresponding to the principal build directions relative to the applied scratch.

The scratch test was conducted according to a testing plan written as a script in the tribometer software version 2.0. Two types of tests with a constant load force or with a linear rising load force were considered. The loading force F_z_ was modified gradually, starting from 5 N and having a maximum force for depending on each test probe. The maximum force value was calculated depending on the tangential force F_x_ during tests, making sure that F_x_ did not exceed the domain of the measuring sensor (20 N). When reaching this value, the sensor enters protection mode against overloading. The testing sequences were the following:

(a) Force that rose dependent on the time;
(b) Constant force over time.

The testing sequences had a fixed time interval of 60 s, and the linear table moved 10 mm, which resulted in a pointer moving speed of 0.167 mm/s.

The measured parameters during the tests were the loading force F_z_, tangential force F_x_, time, and linear movement of the table. Based on those parameters, the following graphs of variation of force and time with movement were made.

**Figure 4:**
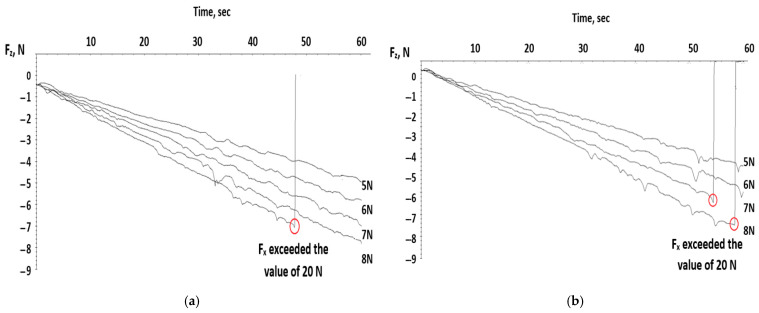

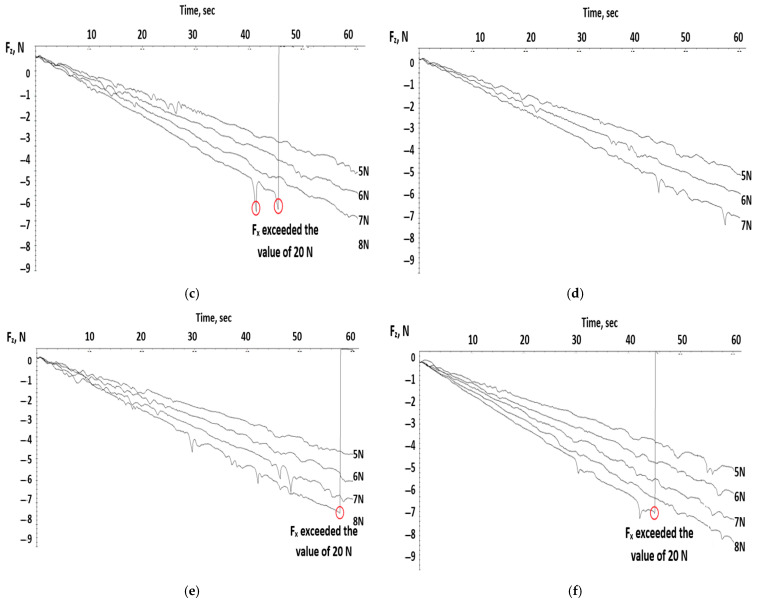
Linear variation in time of loading force for all test probes. (**a**) test probe I_1 (**b**) test probe I_2 (**c**) test probe II_1 (**d**) test probe II_2 (**e**) test probe III_1 (**f**) test probe III_2.

Figure 4 indicates the linear variation in time for the loading force F_z_ for each testing probe. The loading force was modified after each test starting from 5 N with 1 N increments. On these graphs, the points where the test was stopped (equipment reached safe mode and the carrier was retracted) because the sensor limit was reached are visible. This behavior is even better visualized on the graphs below.

**Figure 5:**
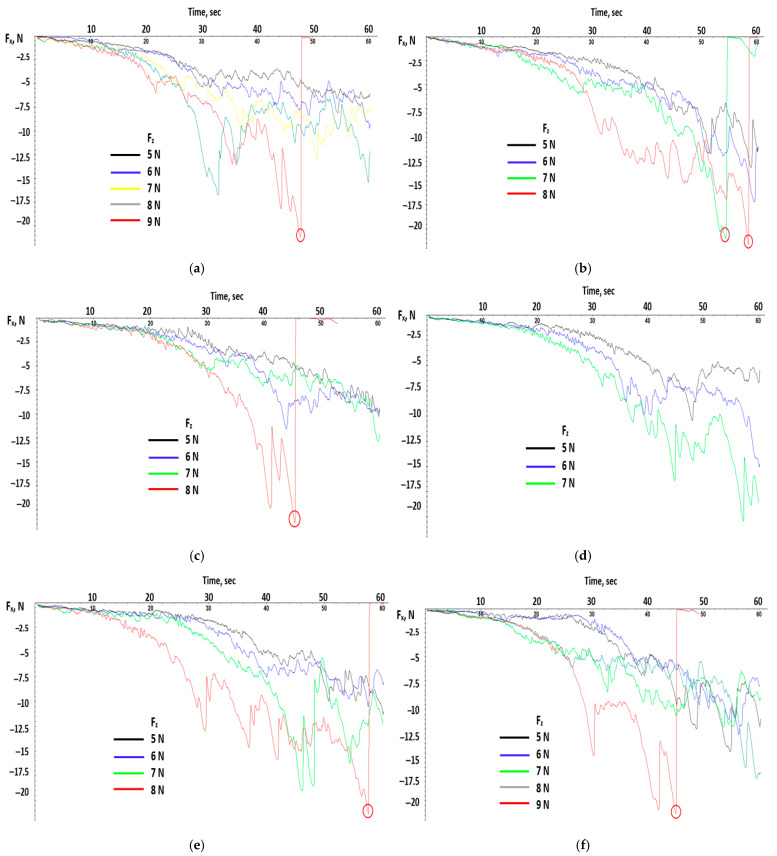
Variation in tangential force over time for all test probes. (**a**) test probe I_1 (**b**) test probe I_2 (**c**) test probe II_1 (**d**) test probe II_2 (**e**) test probe III_1 (**f**) test probe III_2.

The graphs in Figure 5 show, as was expected, a rise in the tangential force F_x_ with loading force F_z_ due to the material resistance at the pointer insertion. This rise has a small slope at the beginning, approximately during the first 30–40 s, where it can be observed that the fluctuations of the tangential force are small and became bigger once the test was about to end. Also, the maximum values of the tangential force could exceed the 20 N value, which made the tribometer stop.

The red circle marks the critical load at which a significant transition in the scratch behavior occurs—specifically, when the normal load exceeds 20 N. At this point, the plastic material begins to exhibit permanent deformation, such as plowing, micro-cracking, or material removal. This threshold is often associated with the onset of ductile-to-brittle transition or a change in failure mechanism. Identifying this critical load is essential for understanding the scratch resistance and durability of the polymer under applied stress.

**Figure 6:**
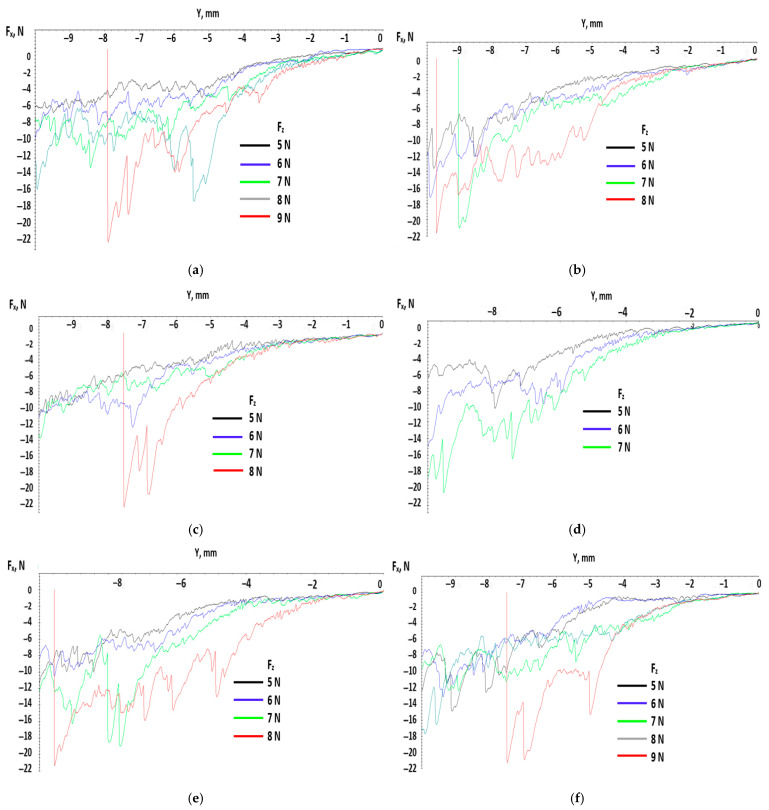
Variation in tangential force with distance for all test probes. (**a**) test probe I_1 (**b**) test probe I_2 (**c**) test probe II_1 (**d**) test probe II_2 (**e**) test probe III_1 (**f**) test probe III_2.

The graphs below from Figure 6 show the same slow variation of the tangential force in the first part of the test for 5–6 mm and bigger variations after that till the tangential force reaches the maximum value.

**Figure 7:**
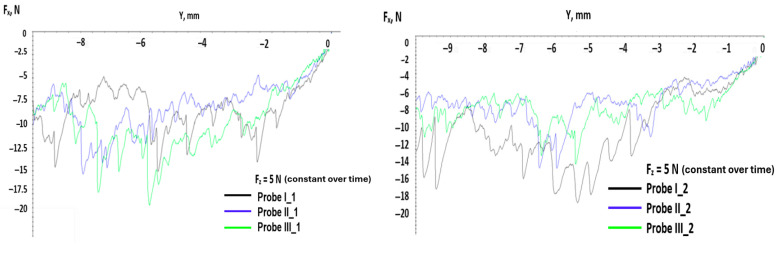
Variation in tangential force with distance at a loading force of 5 N applied constant in time.

Testing with constant force over time means that in the preloading phase, the pointer is pressed against the test probe with a given force and after that, the force is constant for the rest of its movement. This means that the indenter induces localized stress in the material from the beginning, compared with the previous tests where the force rose over time and the tensions from the material rose progressively. It was expected that on the graphs below, the variation in the tangential force produced a big variation due to the protrusion of the pointer in the material of the testing probes. In Figure 7, a small variation for the first 2–3 mm can be observed compared with the cases with linear force over time, where the distance was approximately 5–6 mm.

**Figure 8:**
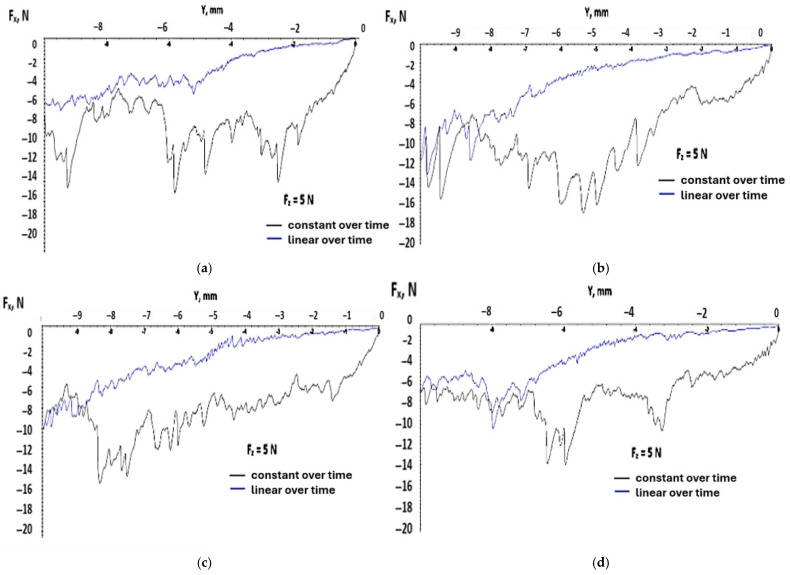

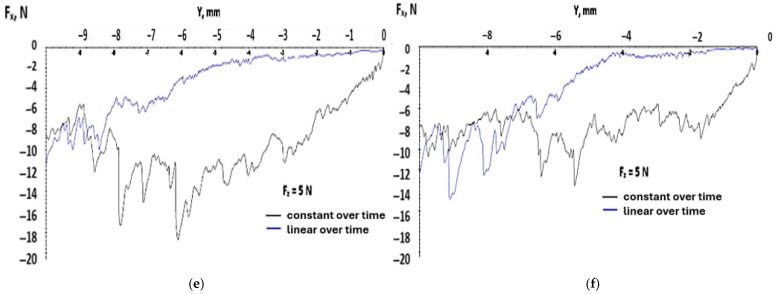
Variation in tangential force with distance for all test probes at loading force F_z_ = 5 N. (**a**) test probe I_1 (**b**) test probe I_2 (**c**) test probe II_1 (**d**) test probe II_2 (**e**) test probe III_1 (**f**) test probe III_2.

Also, for the case with a constant force over time, the rise in the tangential force was abrupt with a big slope compared with the slow rise of the linear force over time. This aspect can be observed in the graphs of Figure 8, which show the variation in the tangential force with distance for each test probe for the two different cases of application for the normal force.

In the scratch tests, the SLA Black V4 resin samples showed varying resistances based on the orientation. The horizontally oriented samples (H) resisted scratching better than the vertical (V) or diagonal (D) samples.

This finding is consistent with the third hypothesis (H3), supporting the claim that layer adhesion is weaker along the Z-axis, a known limitation in photopolymer prints. Surface delamination or cracking initiated more readily in the diagonal and vertical orientations, consistent with the stress concentration at the layer boundaries.

### 3.2. Tensile Test

Knowing the properties of composite materials to be processed through the injection process is of great importance, both for carrying out the injection process under optimal conditions with energy savings and for obtaining quality benchmarks that fully serve the use for which they were created.

By extracting the values from the INSTRON 8801 testing machine, we obtained the characteristic curves of the materials, as determined for each specimen. The different behaviors of the curves were provided by the distribution of fibers/minerals inside each sample.

The test specimens were cut from real-world injection-molded housings made from each PP composite. To assess the influence of the fiber orientation, the samples were extracted in three orientations relative to the injection point: horizontal (T), vertical (L), and diagonal (D).

For each material and orientation, one specimen was prepared (total *n* = 9). All samples were conditioned at 23 ± 2 °C and 50 ± 5% relative humidity for 48 h prior to testing, in accordance with ISO 527-1:2019 [25].

**Figure 9:**
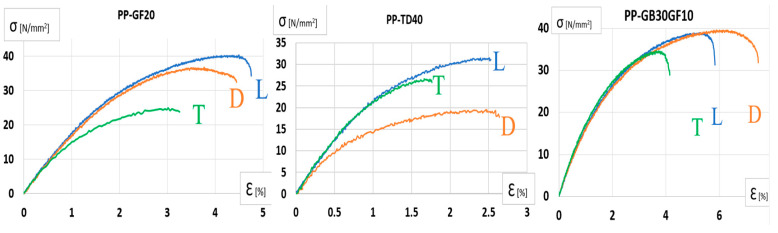
Material characteristic curves for each material.

As we can see from the characteristic curves from Figure 9, the difference between the mineral reinforcement of the plastic material and the glass fiber reinforcement leads to a faster rupture of the specimens, and implicitly, a lower specific elongation. The transition to the plastic range was much faster for the specimens made of the talc-reinforced plastic material, which has a lower dimensional stability. The glass-fiber-reinforced material resists traction better, which is helped by the behavior of the fibers if the traction is applied along the fibers.

**Figure 10:**
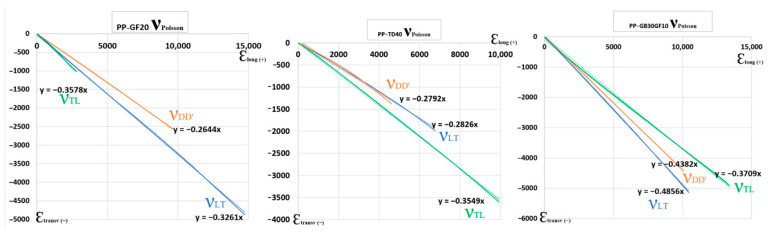
Poisson’s ratio for samples made of each tested material.

**Figure 11:**
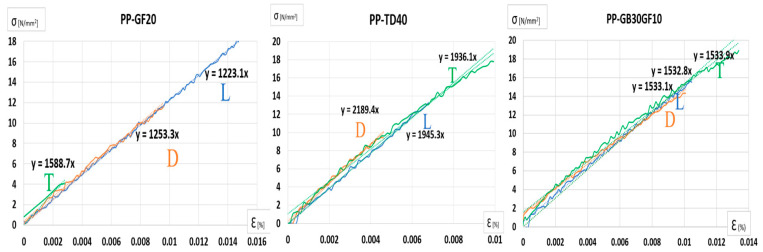
Young’s modulus of each tested material.

The mechanical behavior of the three plastic materials was evaluated through tensile testing in accordance with ISO 527-1:2019 [25]. The results, presented in Figure 10, show the Poisson’s ratios of the materials, highlighting differences in lateral deformation under axial loading. Notably, PP-GF20 exhibited the lowest Poisson’s ratio, indicating limited transverse strain, while PP-GB30GF10 demonstrated a more pronounced lateral response. Complementary to this, Figure 11 illustrates the corresponding Young’s modulus values, providing insight into the stiffness of each material. Among the three, PP-TD40 displayed the highest modulus, suggesting superior resistance to elastic deformation. These findings offer a comparative understanding of the elastic properties critical for structural applications.

## 4. Conclusions

### 4.1. Conclusions for Scratch Tests

Scratch tests made on test samples from an SLA 3D-printed housing with Black V4 resin show a clear anisotropy behavior of friction and deterioration due to the layer orientation against the direction of the pointer pressing force.

For samples with transversal layers, scratches have a deep propagation without big material spin-offs. The surface has a medium scratch resistance with a constant deepness. The layer adhesion was not affected because the pointer-pressing force was parallel with the layers.

For the samples with perpendicular layers, the test showed visible material spin off that was caused by a partial delamination. The resistance to scratching is small, with deep marks and local fractures.

For the test probes with a diagonal layer orientation, the scratch marks are visibly unstable with small digging traces in some areas. In the areas where the pointer meets the layers, local micro-peeling appears. Compared with previous cases, the resistance to scratching is inconstant but without major damage.

Depending on the application, it is recommended to have a layer orientation parallel with the external force in areas where friction or contact wear can appear; this will lead to a lower risk of delamination or material peel off.

### 4.2. Conclusions for Tensile Tests

The orientation of the glass fibers in the outer layers of glass-fiber-reinforced plastics tend to align in the direction of the flow, resulting in a higher tensile strength and stiffness in that direction. In general, fiber-reinforced materials have much higher shrinkage in the cross-flow direction than in the flow direction; in some cases, the cross-flow shrinkage can be two to three times higher. This can also be seen in our graphs.

It is very important to understand that the tensile and impact strength data published in the technical data sheets of thermoplastic materials should be used with caution, as most of the published information is based on test samples without weld lines and with a uniform wall thickness (not as in the tested part models). This is where residual stress caused by uneven cooling of the parts due to different material shrinkage or “frozen in” flow stresses come into play. High levels of residual stress can negatively affect certain mechanical properties, as well as the chemical resistance and dimensional stability.

Other defects present on plastic parts that influence these results are weld lines. The visible lines where the material fronts on the surface of an injected part meet during filling (not very visible on black parts) are called weld lines and cause potential cosmetic defects and reduced mechanical performance. The tensile and impact strengths at the weld line are reduced. The resulting notches at the weld line also act as a stress concentrator, further reducing the impact strength.

The weld line strength in thermoplastic materials varies depending on the resin and injection process parameters, such as the flow front temperature, distance from the injection point, fill pressure, mold pressure, and temperature.

### 4.3. Overall Conclusion

Based on the conducted tests, it is evident that the mechanical performance of polypropylene-based composites is strongly influenced by the type and concentration of fillers, with materials reinforced with glass fibers (such as PP-GF20 and PP-GB30GF10) exhibiting significantly higher stiffness and tensile strength compared with talc-filled variants, like PP-TD40. The orientation of glass fibers, induced by the injection molding process, plays a critical role in enhancing the Young’s modulus along the flow direction, contributing to the anisotropic behavior of the material. Additionally, the scratch test performed on the 3D-printed specimens using Formlabs Black V4 resin highlighted the surface vulnerability of photopolymer materials under localized mechanical stress, underlining the importance of considering the surface resistance alongside bulk mechanical properties in design applications. These findings provide valuable insights for material selection and part design, especially in applications requiring both structural integrity and surface durability.

## 5. Limitations

This study presents a controlled comparative analysis but has certain limitations:

The sample size was limited, particularly in the scratch test, which may affect the generalizability.

The anisotropy from manufacturing—particularly in the injection-molded parts—was not quantitatively characterized (e.g., via microscopy).

The study focused on macromechanical properties; micromechanical failure modes (fiber pullout, matrix cracking) require further investigation.

Future work should include microstructural imaging, larger sample sets, and fatigue or creep testing under real-world loading.

## Figures and Tables

**Table 1:** Summary table of samples.

Label	Material	Graph	Orientation	Sample Source	Notes
I_1	Black V4 resin	(a)	Diagonal	SLA-printed	From same housing, cut 1
I_2	Black V4 resin	(b)	Diagonal	SLA-printed	From same housing, cut 2
II_1	Black V4 resin	(c)	Horizontal	SLA-printed	From same housing, cut 1
II_2	Black V4 resin	(d)	Horizontal	SLA-printed	From same housing, cut 2
III_1	Black V4 resin	(e)	Vertical	SLA-printed	From same housing, cut 1
III_2	Black V4 resin	(f)	Vertical	SLA-printed	From same housing, cut 2
L	PP-GB30GF10, PP-TD40, and PP-GF20		Vertical	Injection-molded	
T	PP-GB30GF10, PP-TD40, and PP-GF20		Horizontal	Injection-molded	
D	PP-GB30GF10, PP-TD40, and PP-GF20		Diagonal	Injection-molded	

## Data Availability

The original contributions presented in this study are included in the article. Further inquiries can be directed to the corresponding author.
